# Selection and Molecular Characterization of Promising Plum Rootstocks (*Prunus cerasifera* L.) among Seedling-Origin Trees

**DOI:** 10.3390/life13071476

**Published:** 2023-06-29

**Authors:** Kubra Korkmaz, Ibrahim Bolat, Aydın Uzun, Muge Sahin, Ozkan Kaya

**Affiliations:** 1 Department of Horticulture, Graduate School of Natural and Applied Sciences, Harran University, Sanlıurfa 63290, Türkiye; 2 Department of Horticulture, Faculty of Agriculture, Harran University, Şanlıurfa 63290, Türkiye; 3 Department of Horticulture, Erciyes University, Kayseri 38030, Türkiye; 4 Republic of Turkey Ministry of Agriculture and Forestry, Aegean Agricultural Research Institute, İzmir 35660, Türkiye; 5 Republic of Turkey Ministry of Agriculture and Forestry, Erzincan Horticultural Research Institute, Erzincan 24060, Türkiye

**Keywords:** *Prunus cerasifera* L., hardwood cutting, rootstock traits, screening, dwarf rootstock

## Abstract

The plum (*Prunus cerasifera* Ehrh) has been used worldwide both as a genetic source for breeding new rootstocks and as clonal rootstock for many *Prunus* species. Considering situations where wild relatives of plums are endangered, in-depth characterization of rootstock traits of genetic diversity of plum germplasm of Turkey with many ecogeographical locations is crucial. In the present study, therefore, three steps were followed for the selection of rootstock candidates among the plum germplasm grown in the Middle Euphrates. This region is characterized by an extremely hot climate with extremely warm summers and very low precipitation in summers. Initially, 79 rootstock candidates were selected based on rootstocks traits, and Myrobalan 29C was also used for the control rootstock in all steps. Hardwood cuttings were taken from each rootstock candidate, and after the rooting process in rootstock candidates, 39 rootstock candidates outperforming other candidates were selected according to root characteristics. Based on rooting ability, forty rootstock candidates with the longest root length below 33.50 mm, root number below 3.00, and rooting cutting number below 30.00% were eliminated. The second step of the study focused on the dwarfing characteristics of 39 rootstock candidates, and 13 and Myrobalan 29C out of 39 rootstock candidates’ dwarfing traits showed value higher compared to the other 26 rootstock candidates. Results indicated that the vigor of rootstock candidates was usually found to be strong (26), intermediate (4), and weak (9). Moreover, 13 out of 39 rootstock candidates’ dwarfism trait was better than the other 26 rootstock candidates. In Step 3, some morphological, physiological, and molecular evaluations were conducted in 13 rootstock candidates and the Myrobalan 29C clone, and there were significant differences between both rootstock candidates and the parameters evaluated. PCA has also been indicated that the reference rootstock Myrobalan 29C was grouped with 63B62, 63B69, and 63B14. The highest genetic similarity was found between 63B11 and 63B16, as well as between 63B76 and 63B66, while the lowest genetic similarity was observed between 63B72 and 63B61 candidates. Overall, the findings presented here provide valuable information about the level of rootstock candidates that could potentially be superior among previously uncharacterized plum cultivars in this plum-growing region of Turkey.

## 1. Introduction

As our world continues to warm up, the reality for many plant species remains ice cold: the average global temperature may be rising; however, this increase is often accompanied by erratic and extreme temperature fluctuations that pose an even further risk of abiotic stress such as drought stress and frost damage to many plants species [1,2,3,4,5]. Predictions of global environmental change have, as a matter of fact, indicated that the extreme increase in air temperature and the consequent effect of global warming have a significant impact on agricultural products by limiting the availability of water and accelerating the occurrence of the drought period [6]. Considering that there will be an increase in both the severity and frequency of drought in the near future, the development and breeding of more productive and drought-adapted plant varieties or rootstocks become more and more important. Therefore, it could be used in efforts to improve the drought tolerance of plants through molecular/genetic approaches, cultural methods, and selection of new rootstocks among seedling-origin trees, and these efforts play a key role in achieving current goals. Much of the research has, in this context, focused on finding out the best strategy to improve the overall performance and yield of plants between extreme and frequent weather events and to overcome the cumulative effects of drought stress on plants [7,8]. Indeed, considering that related or wild progenitor species, landraces, and old traditional cultivars have been cultivated for a long time in reaching these goals, a very important reservoir deficit of new clonal rootstock candidates suitable for breeding purposes may have emerged in the gene pools.

The main purpose of establishing an orchard is to choose the appropriate rootstocks, as well as to choose varieties with good biological and yield characteristics. Rootstock use in fruit growing dates back to ancient times, and many rootstocks were selected from the wild population. In recent years, both modern agriculture and developing techniques have led to an increase in the use of clonal rootstocks, and thus, studies on clonal plum rootstocks draw attention. As a matter of fact, various clonal rootstocks have been developed by some researchers [9,10,11], which are as follows: (I) Myrobalan (*P. cerasifera*), as clonal or seed selections, namely ‘GF31’, ‘H29C’, and ‘B’; (II) Marianna (*P. munsoniana* × *P. cerasifera*), as seed or cloned as ‘Buck’, ‘GF8-1’, and ‘2624’; (III) *P. instititia* cloned selections, including ‘St Julien GF655-2’, ‘St Julien A’, and ‘Pixy’. In additional, although clonal selection or seedlings of Julien are more popular in countries such as England, the Netherlands, and Scandinavia, most of the plums grown are currently produced on seedling rootstocks in many countries—generally Myrobalan seedlings [12]. Myrobalan seedling is currently the most widely used rootstock for plums in Turkey due to being popular among nurserymen [13,14]; however, growers are less satisfied with it because of some disadvantages such as suckering, inferior precocity, too vigorous growth, and significant incompatibility of many cultivars grafted on it [15]. Despite these disadvantages, the main reason why it is the most used as a rootstock in plum cultivation is that it can reduce management costs such as pruning and harvesting in grafted varieties on it and improve production efficiency [16]. These plum rootstocks are, indeed, more tolerant of waterlogging and compact soils than other *Prunus* L. species and also provide greater tolerance to soil-borne pathogens such as root-knot nematodes and fungi and to iron-chlorosis deficiency, which is common in many fruit-growing regions of the Mediterranean area [17,18,19,20,21]. 

Although there are *P. spinosa*, *P. cerasifera*, and *P. domestica* species in different regions of Turkey, the plum variation grown in Turkey generally consists of wild forms (Myrobalan plum, cherry plum, green plum) of *P. cerasifera* Ehrh., which are adapted to the varied soil and climatic conditions within the Asian part of modern-day Turkey [22,23]. Turkey, especially the Mediterranean, Black Sea, Aegean, and the Middle Euphrates regions, has many important green plums (*P. cerasifera* Ehrh.) and genotype diversity [22]. Considering this abundant plum population, different studies have been carried out by some researchers for both rootstock and variety breeding in different regions of Turkey, except for the Middle Euphrates [24,25]. In this regard, plum germplasm accessions of the Middle Euphrates (landraces, related or wild progenitor species, old traditional cultivars, particularly in the original centers of diversity) were less subject to selection pressures. In addition, the weather in this region is very hot in the summer, and the precipitation rate is very low, and in this respect, the potential of finding rootstock candidates that are drought- and high-temperature-tolerant among the plum genotypes grown under these conditions for many years seems to be high [23]. Therefore, this region can play a very important role in the gene pool reservoir that can be used for breeding, especially in the areas of new rootstock selection, disease resistance, fruit quality, and climatic adaptability. Given the strategic role of the region’s plum population both present and future, this germplasm in general deserves special coordinated and attention efforts of conservation, utilization, and evaluation for breeding purposes by both genomic and conventional approaches.

In the near future (especially in the light of the climate change and the physiology of the plum tree), the agronomic characteristics required of the new plum rootstocks could also be different. Therefore, based on all of the above-listed advantages both for the region and for these agronomic traits, we sought a response to the following questions: (i) Are there candidates with superior rootstock characteristics within the existing biodiversity in the plum germplasm grown in the Middle Euphrates?, and (ii) How and to what extent can the identified rootstock candidates be used appropriately in breeding programs or whether they will represent concrete expectations for the future? 

## 2. Materials and Methods

### 2.1. Plant Materials

This study included selections from the autochthonous germplasm, representing the natural populations of *P. cerasifera* Ehrh. (tree ages; 12–16) from the Middle Euphrates, Turkey, in 2020–2022 (Supplementary Figure S1). Surveys were conducted at the experimental field of the Şanlıurfa, located in Halfeti ve Birecik (37° N; 37° E) at 450–525 a.s.l. With the origin of rootstock candidates growing in natural habitats or nature conditions along roadsides, producer orchards grown from seed and field edges were determined by tier numbers (localities in the Middle Euphrates) and different locations, and Myrobalan 29C was also used for the control rootstock. This region is characterized by an extremely hot climate with extremely warm summers and very low precipitation in summers. The soil structure of the region was clayey (30–35%), slightly alkaline (pH; 7.25–7.64), calcareous (28–33% CaCO_3_), and low in organic matter (0.5–0.9%). Climatic values for many years can be found at http://www.secheresse.info/spip.php?article123805 (accessed on 20 May 2023). Based on 54 years of climate data, the annual precipitation average, maximum temperature average (July; 39.7 °C, August; 39.3 °C), and relative humidity were 375.5 mm, 25.5 °C, and 56.5%, respectively [26]. Considering these conditions of the region, the study was carried out in three stages.

### 2.2. In Step 1

Among the rootstock candidates adapted to these climatic conditions, 79 rootstock candidates were determined visually based on some important criteria (weak vigor and no sucker, short internodes, spreading and drooping habitat, fruit set and productivity as well as trees that cannot have negative symptoms against biotic and abiotic stresses) to select genitors for future breeding in 2020. On each harvest day for rootstock candidates, the cuttings from all rootstock candidates were collected in the morning. Then, hardwood cuttings were wrapped in a wet towel and placed in plastic collection bags that were placed in an ice-cold cooler for transport to the Harran University Research laboratory for rooting prepare. The cuttings brought to the laboratory were stored overnight in a walking cooler at 4 °C. The following morning, the cuttings ranging from 5–6 mm in diameter were removed from the cooler and cut uniformly about 24–26 cm lengths with a flat top cut ∼5 mm above the top node and a flat bottom cut ∼5 mm from the lowest node. Cuttings were treated with indole-3-butyric acid (IBA) to avoid any injurious effect of using alcohol as a solvent and due to its high solubility in water. Then, the trial was set up in a randomized complete block design as five replications with 12 plants per replication, where the blocking factors were in Harran University Research greenhouse, and the date the plant material was collected (2021: block 1 and 2, 18 February; blocks 3 and 4, 19 February; blocks 5 and 6, 20 February; block 7 and 8, 21 February). For every block, 60 hardwood cuttings of each rootstock candidate were dipped for 15 s at ∼2 cm depth of the following treatment: a solution of 3000 ppm IBA prepared in reverse osmosis water. Treatment of each rootstock candidate was then planted 10 cm deep in perlite in a randomly assigned location in a 52 cm × 10 cm × 36 cm plastic tray with drainage. The greenhouse thermostat was set to 22–23 °C, and the misting frequency was set to 7 s every 25 min. Cuttings were carefully removed 45 and 60 days after application to collect data on rooting. Cuttings with roots longer than 10 mm long were removed from the experiment and transplanted into pots, but cuttings with roots less than 10 mm long or no roots were replanted in the trays and put back under the mist. In the experiment, the rooting rate of the cuttings and the number of roots were determined by counting, and their root length was measured with a ruler. In this process, based on rooting rate (%), root number, and the longest root length (mm) parameters, 79 rootstock candidates and Myrobalan 29C were screened, and the best 39 rootstock candidates were transferred to the second stage of the study.

### 2.3. In Step 2

On the other hand, 39 rootstock candidates eliminated in the first step of the study were removed from the experiment, and the remaining 39 rootstock candidates and Myrobalan 29C clone were transferred to the second stage in 2021. In step 2, each rootstock candidate was transferred in a 2:1:1 peat: perlite: soil mix in 25 × 25 × 23 cm plastic pots with drainage that had a total volume of 10 L. The trial was set up in a randomized complete block design as five replications with 12 plants per replication, where the blocking factors were in Harran University Research greenhouse. The trail was performed under a greenhouse with a PAR (Photosynthetically Active Radiation) between 650 to 840 mmol m^−2^ s^−1^ and at a relative humidity between 50 ± 5%, as well as at 25 ± 2 °C during the day to 21 ± 2 °C at night. The plots were also watered using a drip irrigation system based on the amount of soil moisture at three-day intervals. Once a month during the season starting in mid-May, all growing rootstocks in pots were watered with a solution, containing macro- and micro-elements: K_2_O—18.0%; P_2_O_5_—6.0%; N—18.0%; Cu—0.010%; Zn—0.07%; B—0.025%; Mn—0.02%; Mo—0.004%; Fe—0.2%.

The following traits of rootstock candidates were evaluated:

Height of the leader (cm)—the leader height was determined by making the last measurement with a ruler after the end of the growing season in the planting year.

Internode height of the leader (mm)—the leader internode height was determined by making the last measurement with a ruler after the end of the growing season in the planting year.

Trunk diameter of the leader (mm)—the measurement was evaluated at 2.5 cm above the base of the shoot, and then the leader diameter was determined by making the last measurement with a caliper after the end of the growing season in the planting year.

### 2.4. In Step 3

Based on the criteria above, dwarf rootstocks were determined from among 39 rootstock candidates, and 13 more dwarfed candidates were transferred to the third step of the study. Myrobalan 29C was also used for the control rootstock in 2022. In step 3, in the next season, pot volumes were increased because the seedlings would develop more depending on the age of the seedlings. Each rootstock candidate was transferred in a 2:1:1 peat: perlite: soil mix in a 32 × 38 × 38 cm plastic pot with drainage that had a total volume of 20 L. The trial was set up in a randomized complete block design as five replications with 12 seedlings per replication, where the blocking factors were in the Harran University Research greenhouse. The plots were also watered using a drip irrigation system based on the amount of soil moisture at three-day intervals. Once a month during the season starting in mid-May, all growing rootstocks in pots were watered with a solution containing the following macro- and micro-elements: K_2_O—18.0%; P_2_O_5_—6.0%; N—18.0%; Cu—0.010%; Zn—0.07%; B—0.025%; Mn—0.02%; Mo—0.004%; Fe—0.2%.

The following traits of rootstock candidates were evaluated:

Leaf area (cm^2^)—in mid-July, three main leaves in the middle of the leader shoot of the rootstock candidates were removed, and the area of leaves was determined using the ImageJ program [27].

Chlorophyll content (Chl)—in mid-July, three main leaves between the third and fifth leaves in the same positions between the apical and middle region of the leader shoot of the rootstock candidates were considered, and Chl content was measured with SPAD-502 Plus (Konica Minolta Optics, Inc., Tokyo, Japan). The content of chlorophyll was determined by averaging the measured values based on the SPAD readings [28].

Stomata conductivity (SC)—in mid-July, measurements performed between 12.00–14.00 h using the Leaf Porometer (Model SC-1 Steady-State Diffusion Porometer, Decagon Devices Inc., Pullman, WA, USA) on the third and fifth leaves in the same positions between the apical and middle region of the leader shoot selected randomly from the rootstock candidates [29]. Results are expressed in mmol m^−2^ s^−1^.

DNA isolation and PCR studies—in early-spring, DNA for rootstock candidates was extracted from young and fresh leaf tissue with cetyltrimethylammonium bromide (CTAB) according to the method stated by Doyle and Doyle [30]. DNA concentrations were measured using the Nanodrop ND 1000 spectrophotometer, and 10 ng/μL DNA solutions were prepared using TE (10-mM Tris–HCl, 0.1-mM ethylene diamine tetra acetic acid). For PCR studies, 16 ISSR primers were tested, and the study was carried out with 12 successful results. PCR components and PCR cycle programs were designed based on the method of Uzun et al. [31]. Then, three μL of loading buffer (20 mL of glycerol (40%), 30 mL of sterile water, and 0.05 g of bromophenol blue) was added to the PCR products, and the mixture was loaded onto a 2% agarose gel. It was then advanced for 3 h under 110 V electric current. Then, 1 × TBE buffer was used to prepare the agarose gel, and 25 μL (0.5 mg/mL) ethidium bromide solution was added to it. Additionally, 100 bp DNA Ladder was loaded as standard in each electrophoresis procedure. After this process, the gels were observed under UV in the imaging system.

### 2.5. Data Analysis

All variables obtained from rootstock candidates were evaluated utilizing the SPSS software (SPSS Version 23); means were compared by Duncan’s test, and differences at *p* ≤ 0.05 were considered significant. On the other hand, PCR bands were scored as absent (0) or present (1) across all 13 rootstock candidates for each primer–pair combination, and the data were utilized to compile a binary data matrix. The MVSP software package Version 3.1 [32] was utilized to calculate Dice [33] similarity coefficients among the rootstock candidates by the formulae Sij = Nij/(Nii + Nij + Njj), where Nii is the number of bands present in the i-th rootstock but absent in the j-th rootstock; Njj is the number of bands present in the j-th genotype but absent in the i-th rootstock; Nij is the number of bands present in both rootstock candidates; and Sij is the similarity index between the i-th and j-th rootstock. Similarity values were clustered by the un-weighted pair-group method of the arithmetic averages UPGMA (Unweighted Pair-Group Method with Arithmetic Average). NTSYS (Numerical Taxonomy Multivariate Analysis System, NTSYS-pc version 2.11, Exeter Software, Setauket, NY, USA, Rohlf, 2000) was utilized to calculate Euclidean distances and construct a dendrogram. Moreover, PCA (Principal Component Analysis) was obtained by JMP (JMP Version 13). HCA (hierarchical clustering analysis) was evaluated by using a heatmap R package (R Version 3.6.3).

## 3. Results

### 3.1. Step 1 in the Selection of Rootstock Candidates

In this step, initial screening was performed based on root traits such as root length, root number, and the number of rooted cuttings of rootstock candidates, and Myrobalan 29C was used for the control rootstock. Our results showed that there were significant differences in the root characteristics of rootstock candidates (*p* ≤ 0.05). The longest root length, number of roots, and number of rooted cuttings of 79 rootstock candidates and Myrobalan 29C ranged from 5 to 100 mm, from 1.60 to 16.32, and from 6.80 to 93.86%, respectively. The 63B78 rootstock candidate had a higher root number than both Myrobalan 29C and other rootstock candidates. The longest root length of 13 rootstock candidates and Myrobalan 29C clone ranged from 67.50 to 100 mm, while root length was less than 60 mm in other rootstock candidates. Similarly, the number of rooted cuttings of 13 rootstock candidates and control ranged from 64.70 to 93.86%, while root length was less than 60% in other rootstock candidates. However, to not overlook rootstock candidates or expand variation, rootstock candidates with root lengths below 33.50 mm, root numbers below 3.00, and rooting cutting numbers below 30.00% were eliminated (Table 1).

### 3.2. Step 2 in the Selection of Rootstock Candidates

At this step, the elimination of rootstock candidates was performed according to characteristics such as the height of the leader, internode height of the leader, and trunk diameter of the leader, which affect dwarf seedlings. Thus, 13 rootstock candidates outperformed the other 26 rootstock candidates with regard to these traits, and they were transferred to Step 3 of the study. Significant differences were observed among the studied rootstock candidates in terms of the characteristics affecting dwarf in seedlings using analysis of variance (*p* ≤ 0.05) (Table 2). The mean number of characteristics dwarfing associated with different rootstock candidates, the height of the leader, internode height of the leader, and trunk diameter of the leader are presented in Table 2. Our results show that 63B16 and 63B66 had the lowest internode height of the leader, followed by 63H61, 63B14, and 63B63. The internode length of the leader among 13 rootstock candidates was lower than Myrobalan 29C. The height of the leader of rootstock candidates ranged from 31 to 97 cm. The studied rootstock candidates showed large differences in height of the leader, so that the vigor of rootstock candidates was usually found to be strong (26), intermediate (4), and weak (9). The range of 3.24–7.20 mm was recorded for the trunk diameter of the leader. The rootstock candidates were clustered into four groups based on the trunk diameter of the leader including extremely narrow (11 rootstock candidates under 4.00 mm), narrow (12 rootstock candidates between 4.00 and 5.00 mm), wide (12 rootstock candidates between 5.00 and 6.00 mm), and wider (5 rootstock candidates between 6.00 and 7.50 mm). In addition, there were significant differences between the 39 rootstock candidates and the Myrobalan 29C in terms of the stem diameter of the leader, and these rootstock candidates had similar stem diameter characteristics (Table 2). In order to see if the findings of the first year showed parallelism with the second year, characteristics such as the height of the leader, internode height of the leader, and trunk diameter of the leader were examined, which affect the dwarf of the seedlings in the second year. Based on our results, dwarfism traits of the same rootstock candidates showed similar characteristics with the data of the first year (Supplementary Table S1).

### 3.3. Step 3 in the Selection of Rootstock Candidates

**Figure 1:**
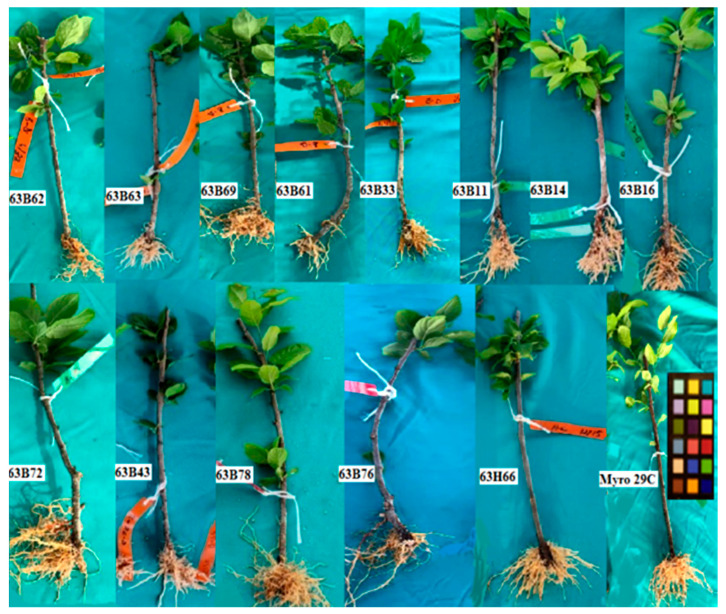
Pictures of rooted cutting of *P. cerasifera* rootstock candidates of Myrobalan 29C studied.

**Figure 2:**
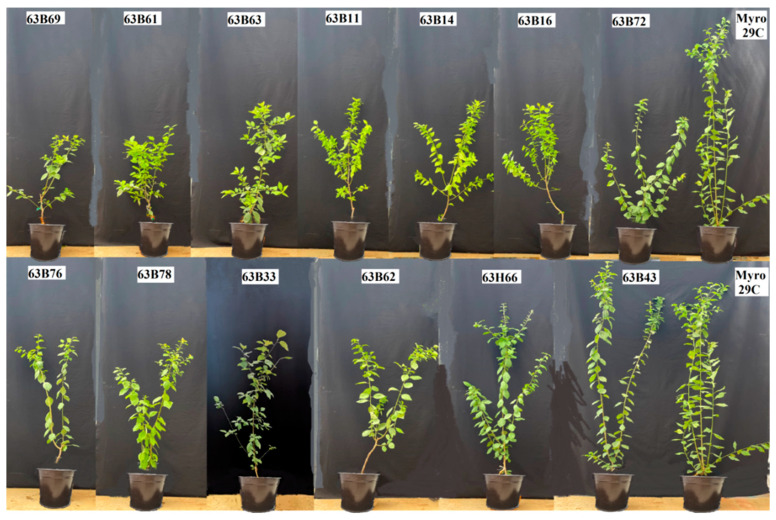
Pictures of grown cutting of *P. cerasifera* rootstock candidates of Myrobalan 29C studied.

**Figure 3:**
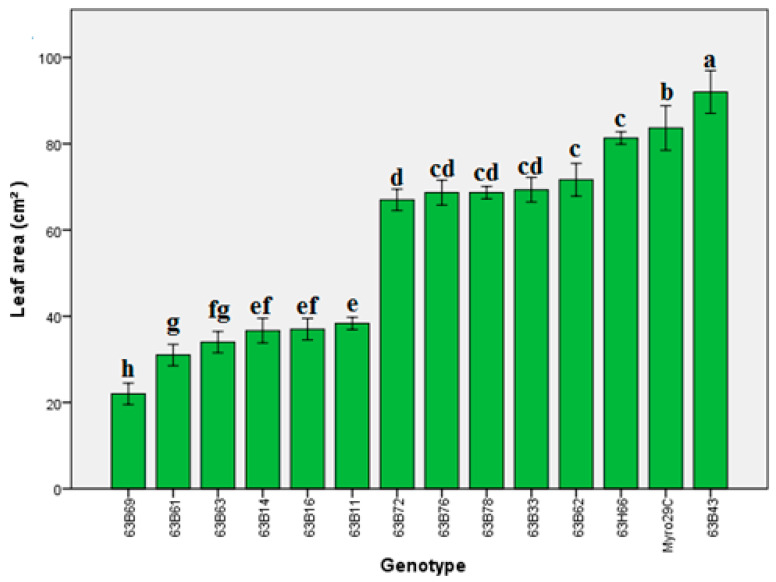
Leaf area of *P. cerasifera* rootstock candidates of Myrobalan 29C studied. Lowercase letters indicate significant differences (Duncan’s test; *p* ≤ 0.05) between applications and stress factors. Error bars on figures represent standard error (SE).

**Figure 4:**
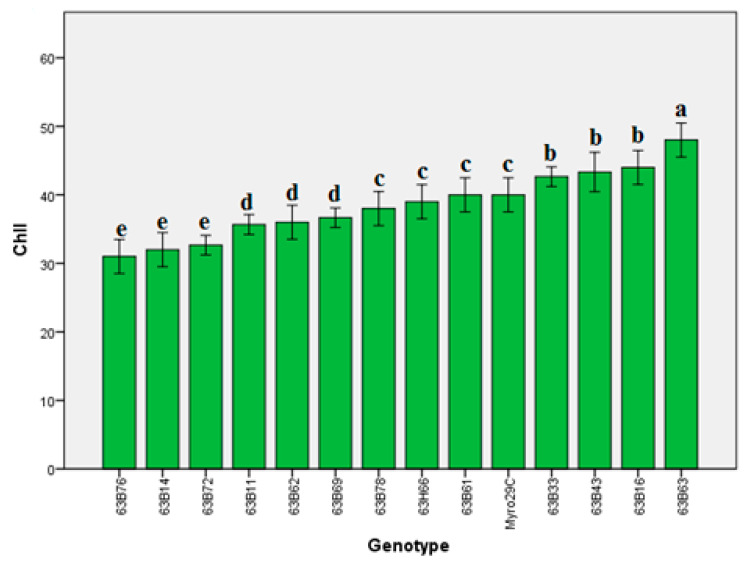
Chl content of *P. cerasifera* rootstock candidates of Myrobalan 29C studied. Lowercase letters indicate significant differences (Duncan’s test; *p* ≤ 0.05) between applications and stress factors. Error bars on figures represent standard error (SE).

**Figure 5:**
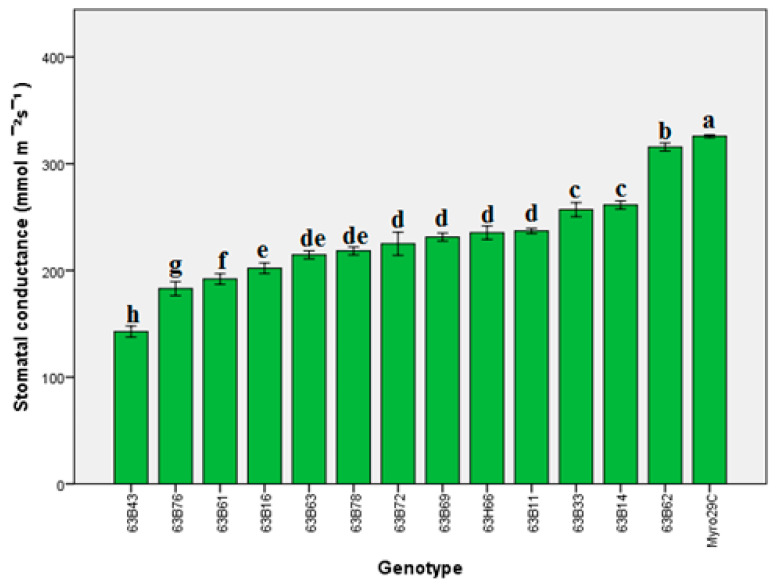
Stomatal conductance content of *P. cerasifera* rootstock candidates of Myrobalan 29C studied. Lowercase letters indicate significant differences (Duncan’s test; *p* ≤ 0.05) between applications and stress factors. Error bars on figures represent standard error (SE).

Our findings indicated that there were significant differences (*p* ≤ 0.05) in leaf area, chlorophyll content (Chl), and stomata conductivity of rootstock candidates (Figure 1, Figure 2, Figure 3, Figure 4 and Figure 5). Values of leaf area ranged among rootstocks from 22.36 to 95.63 cm^2^ (Figure 3), whereas chlorophyll content ranged from 31.83 to 44.16. Compared with Myrobalan 29C, the increases in Chl values were highest in 63B63, 63B16, 63B43, and 63B33, respectively, whereas the Chl value of the remaining 9 rootstock candidates was lower than Myrobalan 29C (Figure 4). One out of 13 studied rootstock candidates and the Myrobalan 29C did show SC value over 300 m^−2^ s^−1^, and the remaining 12 rootstock candidates showed a lower SC value than 250 m^−2^ s^−1^ (Figure 5).

**Figure 6:**
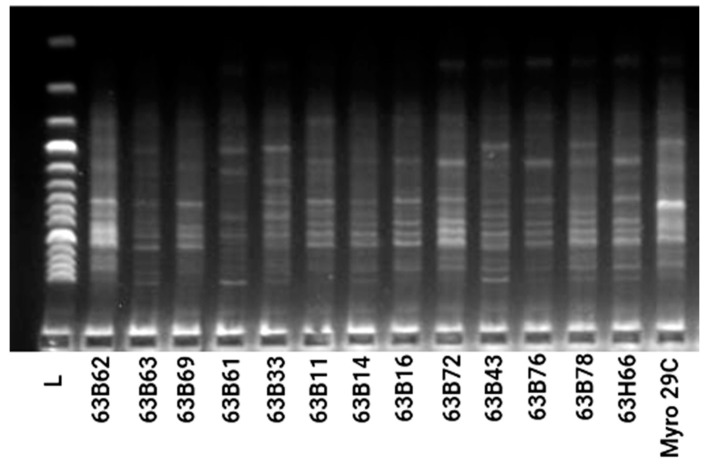
Results of PCR screening with *P. cerasifera* rootstock candidates and Myrobalan 29C ISSR VHVGTG_7_ primer.

**Figure 7:**
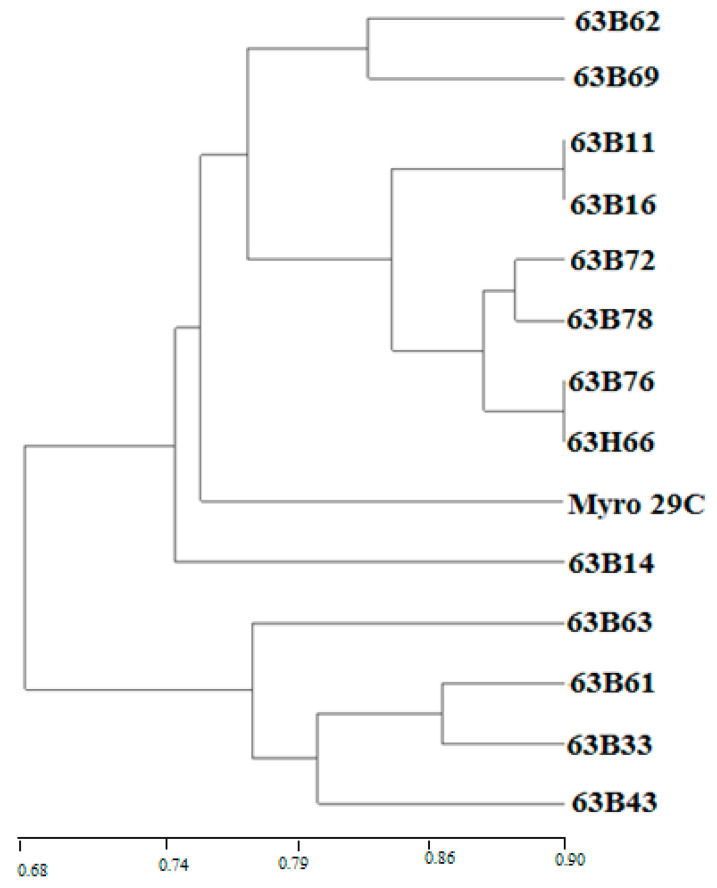
The UPGMA cluster analysis of the ISSR data from plum rootstock candidates based on DICE’s coefficient of genetic similarity.

**Figure 8:**
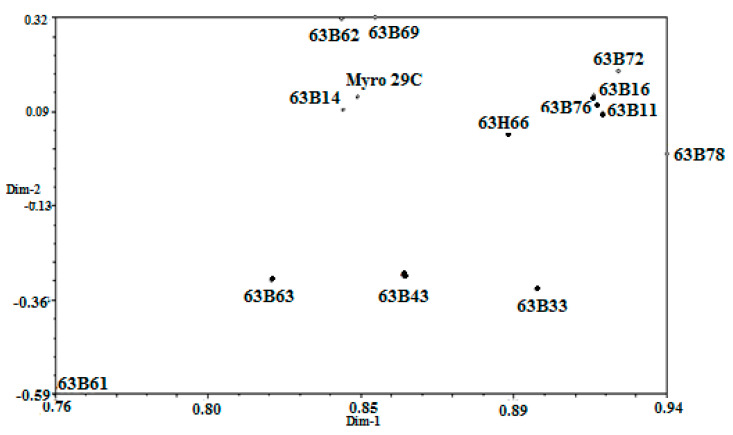
PCA analysis of ISSR data from plum rootstock candidates.

Regarding PCR analysis of genetic diversity in rootstock candidates, all the ISSR markers utilized showed correct amplification and appeared to be polymorphic in the analysis of 13 accessions of plum and one reference rootstock (Table 3). A total of 81 unambiguous selective bands were detected by ISSR fingerprinting of 13 accessions of plum and one reference rootstock using 12 primer combinations which were selected from 16 primer combinations. Among 81 bands, 72 bands were found to be polymorphic, and the mean polymorphic rate was 88.9%. The number of bands obtained from the primers ranged from 15 (VHVGTG7) to 2 (TCC5RY) and all bands were polymorphic in the primers, except for four primers (D8 DACA7, GACA4, CAC3GC, and B8 BCA7C) (Table 3). On other hand, the genetic similarity coefficients of 13 rootstock accessions and the Myrobalan 29C calculated based on Dice (1945) ranged from 0.68 to 0.90 (Figure 6). The highest genetic similarity was found between 63B11 and 63B16, as well as between 63B76 and 63B66, while the lowest genetic similarity was observed between 63B72 and 63B61. Cluster analysis using ISSR data revealed two main groups of rootstock candidates. One of the groups included four rootstock candidates (63B63, 63B61, 63B33, and 63B43), whereas other rootstock candidates were the second of the groups. The first group was divided into two main subgroups (63B14 and others), while the second group was similarly divided into two main subgroups (63B63 and others). The Myrobalan 29C in the first subgroup formed a different group from the other eight rootstock candidates (63B62, 63B69, 63B11, 63B72, 63B78, 63B76, and 63H66) (Figure 7). On the other hand, the PCA from ISSR data produced a similar picture to that given by cluster analysis. The first three eigenvalues on the PCA explained 86.51% of the total variation. The 63B63, 63B61, 63B33, and 63B43 rootstock candidates were significantly different from the others. In addition, 63B62, 63B69, 63B14, and Myrobalan 29C formed a similar group, while 63B72, 63B16, 63B11, 63B76, 63B78, and 63H66 also formed a similar group (Figure 8).

**Figure 9:**
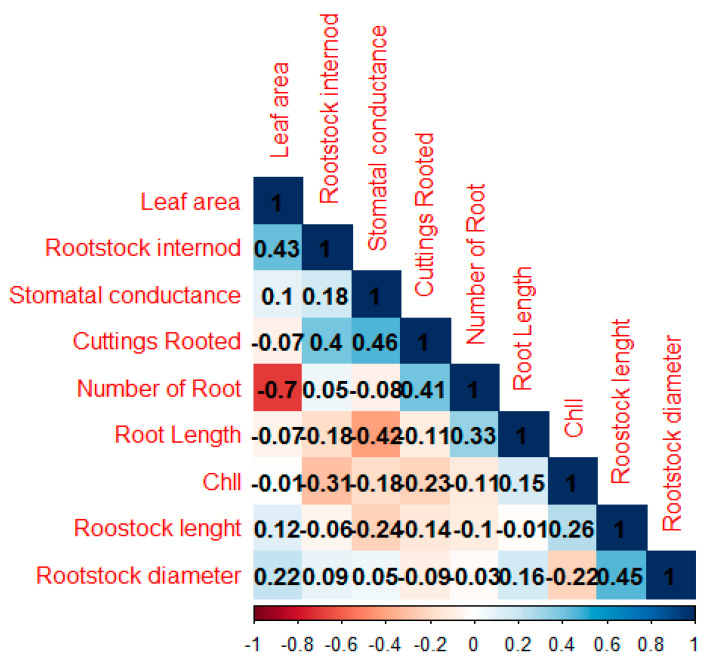
Pearson correlation (*p*-value ≤ 0.05) across all features, where correlation coefficients are indicated by color (dark blue to white indicates a positive correlation 0 to 1, and white to red indicates a negative correlation 0 to −1).

**Figure 10:**
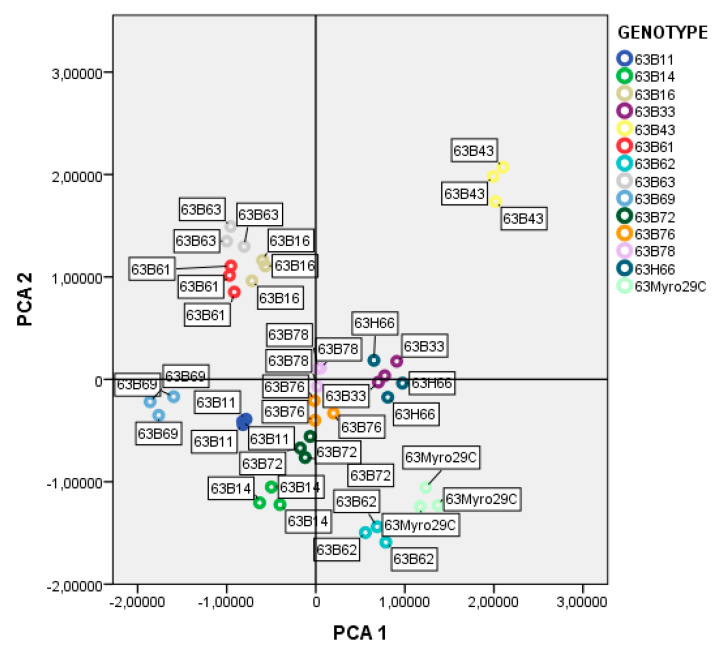
Loading plot of all measured variables included in PCA for rooting parameters some growth parameters, morphological and physiological variables of *P. cerasifera* rootstock candidates, and Myrobalan 29C.

**Figure 11:**
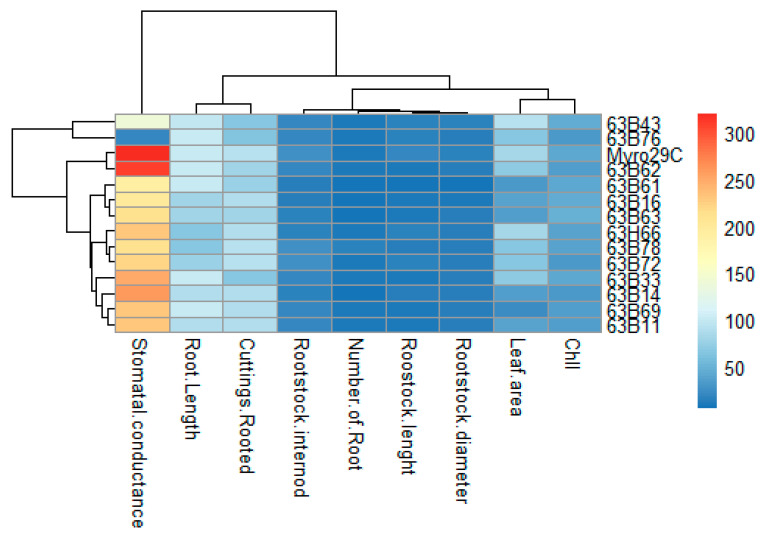
Loading plot of all measured variables included in Heatmap for rooting parameters, some growth parameters, morphological and physiological variables of *P. cerasifera* rootstock candidates.

Although similarities and differences were identified in rootstock candidates with the ISSR method, the correlation between some morphological and physiological evaluations was shown in Figure 9. Correlation analysis indicated negative and positive relationships between parameters, and cuttings rooted were positively and significantly correlated with leaf area (r = 0.43), rootstock internode (r = 0.40), and stomatal conductance (r = 0.46). Rootstock diameter was positively correlated with leaf area, rootstock internode, stomata conductance, rootstock length, and root length, whereas it was negatively correlated with Chl, root number, and cuttings rooted. The number of roots showed a negative and significant correlation with leaf area (r = −0.70) and stomata conductance (r = −0.08). Similarly, Chl showed a negative correlation with leaf area, rootstock internode, stomatal conductance, and cuttings rooted (Figure 10). PCA was, on the other hand, used for an orthogonal transformation to convert a set of observations of parameters measured between rootstocks and possibly related variables into a set of values of linearly unrelated variables. Based on PCA, in the I region, 63B43 formed a stand-alone group, as well as 63B33, 63H66, 63B62, and Myrobalan 29C clustered in the same group within the II region. In addition, 63B14, 63B11, 63B72, and 63B69 clustered in the same group within the III regions, and 63B63, 63B16, and 63B61 clustered in the same group within the VI region, but 63B78 and 63B76 clustered in the origin center (Figure 11). Regarding Heatmap analysis, 63B43 and 63B76 rootstock candidates were in the same group, while other rootstocks formed a different group. Unlike the other rootstock candidates, Myrobalan 29C and 63B62 showed similarities among themselves. In addition, stomata conductance, unlike other parameters, formed a single group, while other parameters formed a separate group among themselves (Figure 11).

## 4. Discussion

### 4.1. Step 1 in the Selection of Rootstock Candidates

Although rootstock breeding started with Myrobalan clones (*P. cerasifera*) in the 1950s, wild plums are of particular interest in rootstock breeding as donors of ecological compatibility or resistance [34,35]. It has been reported that *P. cerasifera* shows the greatest diversity among all *Prunus* species in terms of both ecological adaptation and morphology [14]. Indeed, since related or wild progenitor species, landraces, and old traditional cul-tivars have been cultivated for a long time, a very important reservoir may have been re-vealed in the gene pool that can be used for breeding purposes in the areas of new rootstock selection, fruit quality, climate adaptation, and disease resistance [13,14]. Therefore, *P. cerasifera* is of interest due to its early maturation, disease, good productivity, drought and heat resistance, and tolerance to adverse conditions [23]. Trees grafted on this rootstock usually show strong and vigorous vegetative growth, and microbalances are mostly utilized as seedling rootstocks in fruit growing because they are not root suckers [11,34]. Considering the present and future strategic role of Myrobalan 29C in addition to all these positive features listed above, germplasms rich in wild forms of *P. cerasifera* deserves generally coordinated efforts and special attention to conservation, utilization, and evaluation for rootstock breeding purposes with both innovative approaches and traditional. In this regard, plum germplasm accessions of the Middle Euphrates in Turkey were less subject to selection pressures, and the plum variation has grown generally consisting of wild forms of *P. cerasifera* Ehrh. Therefore, 79 rootstock candidates were determined visually based on some important criteria such as weak vigor, short internodes, spreading and drooping habitat, fruit set and productivity as well as trees that cannot have negative symptoms against biotic and abiotic stresses in this study. After screening in the first year, cuttings were taken from each rootstock candidate in the next season, and their rooting characteristics were determined. There were significant differences in root length, number of roots, and number of rooted cuttings of 79 rootstock candidates, and no rooting occurred in seven rootstock candidates. It has been stated that rooting ability should be considered as an important feature in the first years of the rootstock selection program [34]. It has been, however, reported that *P. cerasifera* is usually propagated by hardwood cuttings and auxin applications between 3000 and 5000 mg/L for dormant cuttings of Myrobalan B’, and other *P. cerasifera* genotypes give the best results on rooting [36,37]. In the present study, therefore, 3000 mg/L auxin was used for dormant cuttings of rootstock candidates, which is consistent with most other previous literature on rooting of cuttings [37,38]. Within all rootstock candidates, cuttings of 63B78 rootstock candidates rooted considerably better than the others. Furthermore, 63B78, 63B33, Myrobalan 29C, 63B43, 63H66, 63B62, 63B76, 63B14, 63B72, 63B69, 63B14, 63B63, 63B16, and 63B61 had the highest root number compared to 66 rootstock candidates. The longest root length and number of rooted cuttings of 13 rootstock candidates ranged from 67.50 to 100 mm and from 64.70 to 93.86%, respectively, while these values were less than 60 mm in other rootstock candidates (Table 1). It is worth mentioning that this difference between the rooting characteristics of rootstock candidates is an expected situation, which is consistent with previous results regarding the rooting characteristics of plum rootstock candidates [39,40]. Therefore, based on rooting characteristics, 39 rootstock candidates with the longest root length below 33.50 mm, root number below 3.00, and rooting cutting number below 30.00% were eliminated in step 1 (Table 1).

### 4.2. Step 2 in the Selection of Rootstock Candidates

The number and quality of flower buds produced on trees increase, as trees on dwarf rootstocks both stop shoot growth earlier and encourage the tree’s assimilation and nutrients to be directed towards the production of flower buds [41,42]. In addition, dwarf trees not only have environmental benefits but also minimize spray targeting and spray drift, as well as the cost per unit of quality fruit produced, facilitating improved resource productivity or mainly labor [41,42]. Based on the positive characteristics of the dwarf rootstocks listed above, the second step of the study focused on the dwarfing characteristics of 39 rootstock candidates. Significant differences (*p* ≤ 0.05) were observed among the studied rootstock candidates regarding the height of the leader, internode height of the leader, and trunk diameter of the leader affecting dwarf in seedlings. In addition, 63B16, 63B61, 63H66, 63B14, and 63B63 had the lowest internode height of the leader compared to other rootstock candidates and Myrobalan 29C. Findings indicated that 13 out of 39 rootstock candidates’ internode traits measured showed a value smaller compared to the other 26 rootstock candidates (Table 2). In addition, our results for the second year were in agreement with the findings for dwarfism traits for the first year (Supplementary Table S1).

The height of the leader and trunk diameter of the leader outperformed the other candidates when 13 rootstock candidates are evaluated in terms of dwarfing characteristics in parallel with the shortness of the internodes. This may be explained by the shortening of the leader in the shoots of dwarf trees and the thickening of the trunk diameter of the leader. However, there is no easy explanation for rootstock-induced dwarfing or rootstock size control, which is a complex trait affected by environmental conditions and growth parameters [43]. Dwarfing in some rootstock candidates in our study, in turn, is likely to be partially attributable to negative environmental conditions and soil properties affecting the growth of trees in the region of the selection. Indeed, the selection region has a climate that receives very little precipitation in the summer and is extremely hot, and its soil structure is very calcareous; the clay content is high, and the organic matter content is very weak. This assumption was consistent with the development of the height of the leader, the internode height of the leader, and the trunk diameter of the leader in dwarf rootstocks, which might explain adverse environmental and growing conditions affecting shoot behavior. Many authors have also interpreted dwarfism to be a complex trait coordinated by the biosynthesis and signal transduction pathway of phytohormones such as brassinosteroids (BRs), gibberellins (GAs), and auxin (IAA) [44,45,46,47]. 

Possible explanations of the difference in dwarfism traits among these rootstock candidates could be variables in the biosynthesis of phytohormones and signal transduction pathway processes depending on the environment and growing conditions. It has also been noted that dwarfism is usually caused by mutations in genes involved in the signaling pathways and biosynthesis of IAA, BR, GA, and strigolactone (SL) that affect plant internode length by regulating cell expansion [48]. Previous studies have, indeed, shown that BR biosynthesis is essential for cell differentiation and cell division, while GAs have significant effects on shoot elongation [48,49]. Adverse environmental and growing conditions are likely to have an impact on tree physiology and biosynthesis of phytohormones—in particular, canopy size, light distribution within the canopies, and light use efficiency—so that smaller and more open canopies with large vertical gaps between long branches may have occurred in the natural environment of dwarf rootstock candidates.

### 4.3. Step 3 in the Selection of Rootstock Candidates

In this step, leaf area, Chl, SC, and molecular studies were evaluated in 13 rootstock candidates and Myrobalan 29C that were better than other candidates with regard to dwarfing properties in step 2. Regarding the leaf area of rootstock candidates, 63B43 had the best results compared to the other candidates, followed by Myrobalan 29C, 63B66, 63B62, 63B33, 63B78, 63B76, and 63B72 (Figure 3). Considering that leaf area plays a role in some physiological processes (photosynthesis, transpiration, absorption, etc.) [50], it is possible that differences in leaf area and size in prominent rootstock candidates may be effective in plant development and yield. Some authors confirmed our hypotheses and clearly demonstrated how differences in leaf area and shape between genotypes within a species affect plant growth and yield [51,52].

The increases in Chl values were highest in 63B63, 63B16, 63B43, and 63B33, respectively, compared with Myrobalan 29C, whereas the Chl value of the remaining 9 rootstock candidates was lower than Myrobalan 29C (Figure 4). Das-Neves et al. [53] stated that SPAD value in *Prunus* species could be used as a reliable method in the evaluation and estimation of grafting incompatibility, although we did not evaluate it at this stage. It was also noted that there is a close correlation between the leaf chlorophyll concentration and the N content of the leaf since most of the leaf chlorophyll molecules contain N [54]. Therefore, these results were promising as a tool to detect and correct N deficiency in plants due to the relationship between Chl content and photosynthetic pigments, which is similar to the results of previous authors [55,56]. 

The highest stomatal conductance was determined in Myrobalan 29C, and the lowest was in the 63B43 rootstock candidate (Figure 5). Based on the report that stomatal transpiration plays a key role in the adaptation of plants to different ecological conditions and tolerance to adverse environmental conditions [57], the knowledge of SC values of rootstock candidates in different ecologies and under different conditions may be of great importance. It has, indeed, been reported in previous reports that there is a close relationship between the density and movement of stomata on the leaves of rootstocks used for fruit species and varieties and the water lost by the plant and the plant–water balance [58]. However, it is also known that many internal and external factors such as light intensity, low CO_2_ concentration, humidity, and ABA affect the density and movement of stomata in leaves [59,60]. Based on these combined findings, we assume that these differences between rootstock candidates in terms of SC values may be influenced by many factors.

ISSR has been noted as a cost-effective technique for the detection of DNA polymorphisms and the identification of plum genetic resources [61,62]. The percentage of polymorphic bands (PPB), Shannon’s information index (I), and Nei’s gene diversity index (h) are important criteria for determining genetic diversity [63]. Our work indicates that the 13 rootstock candidates and Myrobalan 29C clone are genetically diverse as the values of I, h, and PPB are equal to 0.90, 0.68, and 88.9%, respectively. Except for four primers including DBDACA7, GACA4, CAC3GC, and BDBCA7C, the number of bands obtained from the primers ranged from 15 (VHVGTG7) to 2 (TCC5RY), and all bands were polymorphic in the primers (Table 3). These results are higher than those stated by previous authors (I = 0.27, h = 0.15, PPB = 88.4%), which were evaluated utilizing ISSR, and greater than the 26 genotypes in Greece (PPB = 77.33%) determined by both RAPD and ISSR [62,64]. In addition, the relatively low genetic diversity observed in rootstock candidates compared with results among *P. cerasifera* genotypes or derivatives/hybrids studied in other studies is generally consistent with ISSR diversity values observed in vegetatively produced tree genotypes selected from different regions [65,66,67,68]. Regarding cluster analysis, the highest genetic similarity was found between 63B11 and 63B16, as well as between 63B76 and 63B66, while the lowest genetic similarity was observed between 63B72 and 63B61 (Figure 7). The relative genetic diversity of rootstock candidates from the Middle Euphrates could be due to the fact that this region indicates clear ecological differences with regard to rainfall, humidity, average temperature, and soil structure from the region where each tree grows. Some authors confirmed that clear ecological differences in precipitation, humidity, average temperature, and soil structure from the region where each tree grows cause genetic differences [22]. 

The rootstock candidates according to PCA were allocated into four groups, and group 1 contained four rootstock candidates, including 63B63, 63B61, 63B33, and 63B43. In group 2, the reference rootstock Myrobalan 29C was grouped with 63B62, 63B69, and 63B14, whereas 63B72, 63B16, 63B11, 63B76, 63B78, and 63H66 also formed a similar group in group 3. Finally, group 4 was formed exclusively by the 63B61 rootstock candidate (Figure 8). These results suggest that genetically predisposed rootstock candidates cluster together, which is supported by the findings that previously genetically similar individuals often clustered together [69]. In addition, the constant and natural selection pressure of rootstock candidates within the same region seems to be an important factor for genetic adaptation. The relationship between environmental factors and genotypic differentiation in rootstock candidates was also observed via isozyme, morphological and molecular markers, which is in line with the results reported by previous authors [70,71].

Regarding the correlation between some morphological and physiological evaluations in our results, cuttings rooted were positively and significantly correlated with rootstock internode (r = 0.40), leaf area (r = 0.43), and stomatal conductance (r = 0.46). Rootstock diameter was positively correlated with leaf area, stomata conductance, rootstock internode, rootstock length, and root length, whereas it was negatively correlated with Chl, root number, and cuttings rooted (Figure 9). These results are to be expected given that leaves and their area play a key role in some physiological processes such as photosynthesis, transpiration, and absorption. In our results, there were, indeed, significant differences in some morphological and physiological parameters such as stomata conductance, rootstock internode, rootstock length and root length, Chl, SC, root number, and cuttings rooted of rootstock candidates that leaf area and size differences significantly affected plant growth, which was consistent with the results of previous authors [50,51,52,53,54,55,56,57]. 

Considering our PCA results used for an orthogonal transformation to convert a set of parameter observations measured between rootstocks and related variables to a set of linearly unrelated variables, rootstock candidates formed five groups as follows: 63B43 formed a stand-alone group in the I region; 63B33, 63H66, 63B62, and Myro 29C clustered in the same group within the II region; 63B14, 63B11, 63B72, and 63B69 clustered in the same group within the III region; 63B63, 63B16, and 63B61 clustered in the same group within the VI region; and 63B78 and 63B76 clustered in the origin center (Figure 10). This grouping or differentiation could be attributed to environmental and regional differences as well as several factors: (I) this may be relevant to pollination biology as plums cannot fruit parthenocarpically and require cross-pollination due to high self-incompatibility [63]. (II) Based on the pollen being carried by insects and the optimum time for pollination being 1–3 days at the time of flower opening [72], this may be due to the difference in chilling requirements for the flowering of rootstock candidates. Heatmap analysis also supported this assumption, and it indicated that 63B43 and 63B76 rootstock candidates were in the same group, whereas other rootstocks formed a different group. Unlike the other rootstock candidates, Myrobalan 29C and 63B62 showed similarities among themselves. Unlike other parameters, stomata conductance formed a single group, while other parameters formed a separate group among themselves (Figure 11).

## 5. Conclusions

The use of rootstocks is an essential component of modern agriculture, as current global agricultural challenges such as climate change and abiotic and biotic stress conditions imply the need to create new technologies and farming systems. Most of the rootstocks currently used are clonally propagated, and although there are several ongoing efforts to improve new rootstocks, there are generally fewer rootstock breeding programs for scion varieties than for the large number of breeding programs. Breeding programs that will respond to existing fruit production require rootstock-incorporating features including compatibility with a wide number of varieties, low vigor, and tolerance or resistance to abiotic (water stress, hypoxia, drought, salinity, and Fe chlorosis) and biotic (nematodes and diseases) stresses. In this sense, the selection of new clonal plum rootstocks in the Middle Euphrates region of Turkey offers an alternative new rootstocks to *Prunus* spp. In this regard, three steps were followed for the selection of rootstock candidates in the region, and the results of the study highlight that 13 rootstock candidates were promising as compared to Myrobolan 29C. However, since some of our studies on rootstock/scion interaction, the combination of rootstock variety and other *Prunus* species, micropropagation, and abiotic and biotic stress factors of 13 rootstock candidates are still ongoing or will continue, no recommendation has been conducted among them yet. The knowledge on rootstock/scion interaction, micropropagation, and abiotic and biotic stress evaluations of the 13 rootstock candidates may be useful as parents in future breeding programs or to select suitable ones to be grown in different climatic conditions.

## Figures and Tables

**Table 1:** List of the plum rootstock candidates used in this study, their longest root length, number of roots, and cuttings rooted.

Number of Rootstock Candidates	Rootstock Candidates	Root Length (mm)	Number of Roots	Cuttings Rooted (%)
1	63B78	67.50 d	16.32 a	93.86 a
2	63B72	77.50 d	9.40 cd	92.40 a
3	Myrobalan 29C	100 a	11.60 b	92.40 a
4	63B69	100 a	9.00 e	90.33 a
5	63H66	67.50 d	10.00 cd	89.46 a
6	63B11	90.00 b	9.23 d	88.06 a
7	63B14	87.5 c	7.66 e	88.06 a
8	63B16	80.00 c	7.00 e	85.30 ab
9	63B62	100 a	10.00 cd	78.66 bc
10	63B63	80.00 c	7.80 e	78.60 bc
11	63B61	100 a	5.40 f	75.63 cd
12	63B33	100 a	11.80 b	68.90 de
13	63B43	99.66 b	10.26 c	68.16 d–f
14	63B76	100 a	10.00 cd	64.70 ef
15	63H65	57.50 e	4.66 fg	59.30 fg
16	63B68	57.50 e	4.66 fg	53.60 gh
17	63B60	57.50 e	4.33 gh	51.83 hi
18	63B66	57.50 e	4.33 gh	48.16 h–j
19	63B13	52.50 ef	4.33 gh	46.83 i–k
20	63B34	52.50 ef	4.33 gh	46.16 i–l
21	63B77	45.00 gh	4.00 c–h	41.66 i–l
22	63B70	47.50 fg	2.00 e–h	40.03 j–m
23	63H30	45.00 g–i	3.66 g–i	39.80 j–n
24	63H39	45.00 g–i	3.66 g–i	39.13 j–n
25	63B45	45.00 g–i	2.00 e–h	38.86 k–o
26	63H46	42.66 g–i	4.33 gh	38.83 k–p
27	63B19	42.50 g–i	3.66 g–i	36.56 k–q
28	63B12	41.33 h–k	3.66 g–i	35.77 k–r
29	63B56	40.00 i–l	3.66 g–i	34.80 l–s
30	63B42	39.00 i–m	3.66 g–i	33.40 l–s
31	63B53	38.33 j–n	3.66 g–i	31.93 l–t
32	63B47	36.00 j–n	2.40 e–h	31.80 l–u
33	63B54	35.00 k–o	2.40 e–h	31.60 l–u
34	63H27	35.00 k–o	3.00 d–h	31.46 m–v
35	63B46	34.00 l–p	2.30 e–h	31.20 m–w
36	63B51	34.00 l–p	2.15 e–h	30.53 m–x
37	63B73	34.00 l–p	2.00 e–h	30.40 n–x
38	63H35	34.00 l–p	2.00 e–h	30.20 n–x
39	63B15	33.66 l–q	2.00 e–h	30.06 n–x
40	63B18	33.66 l–q	3.00 d–h	30.00 o–x
41	63B55	33.00 m–r	2.66 j–o	29.90 o–x
42	63H40	32.50 n–s	3.33 h–k	29.80 o–y
43	63B49	32.00 n–t	3.00 d–h	29.53 o–y
44	63H26	30.00 o–u	3.33 h–k	28.80 o–y
45	63H60	42.66 g–i	3.10 d–h	28.40 o–z
46	63H36	29.00 p–v	2.00 e–h	28.36 p–a^1^ *
47	63B77	29.00 p–v	3.33 h–k	27.93 q–a^1^
48	63B75	28.33 q–w	2.66 j–o	26.93 r–a^1^
49	63H28	28.00 r–w	2.66 j–o	26.26 r–a^1^
50	63H55	28.00 r–w	2.66 j–o	25.73 r–a^1^
51	63H37	28.00 r–w	2.66 j–o	25.60 s–b^1^
52	63B70	28.00 r–w	2.66 j–o	24.66 s–b^1^
53	63B50	27.50 s–x	2.66 j–o	23.66 s–b^1^
54	63B65	27.00 t–x	2.66 j–o	23.06 t–b^1^
55	63B36	27.00 t–x	2.66 j–o	23.00 u–b^1^
56	63B74	26.33 u–y	2.63 k–q	22.83 v–c^1^
57	63H45	25.00 u–z	2.46 k–r	22.63 w–d^1^
58	63B71	25.00 u–z	2.46 k–r	22.13 x–d^1^
59	63B66	24.00 v–a^1^*	2.33 k–s	21.46 x–d^1^
60	63H34	23.33 w–a^1^	2.33 k–s	21.40 y–d^1^
61	63B64	22.50 x–b^1^	2.33 k–s	21.33 y–d^1^
62	63H54	22.50 x–b^1^	2.33 k–s	20.13 z–d^1^
63	63B32	22.50 x–b^1^	2.33 k–s	20.00 z–d^1^
64	63H22	22.50 x–b^1^	2.33 k–s	19.36 a^1^–d^1^
65	63B79	21.33 y–c^1^	2.30 l–s	19.33 b^1^–e^1^
66	63B68	20.00 z–d^1^	2.00 l–t	18.66 b^1^–e^1^
67	63B31	19.00 a^1^–d^1^	1.93 m–u	15.53 c^1^–e^1^
68	63H44	17.50 b^1^–d^1^	1.93 m–u	14.96 d^1^ e^1^
69	63B48	16.33 c^1^–e^1^	1.83 n–v	13.40 e^1^ f^1^
70	63B67	14.66 d^1^–f^1^	1.66 o–w	12.53 e^1^ f^1^
71	63H41	11.66 e^1^–f^1^	1.60 p–x	7.86 e^1^ f^1^
72	63B48	10.33 f^1^–g^1^	1.60 p–x	7.80 e^1^ f^1^
73	63B67	5.00 g^1^–h^1^	1.60 p–x	6.80 e^1^ f^1^
74	63H41	0 h^1^	0 a^1^ *	0 f^1^
75	63B30	0 h^1^	0 a^1^	0 f^1^
76	63B44	0 h^1^	0 a^1^	0 f^1^
77	63B20	0 h^1^	0 a^1^	0 f^1^
78	63B21	0 h^1^	0 a^1^	0 f^1^
79	63B22	0 h^1^	0 a^1^	0 f^1^
80	63B23	0 h^1^	0 a^1^	0 f^1^

Values marked with the same small letter do differ significantly based on Duncan’s test (*p ≤* 0.05). * To determine the differences between the means, exponential in letters combinations starting from the letter again starting with the letter “a” (as a^1^…h^1^) were used in the comparisons, since the alphabetic letters were insufficient in the comparisons.

**Table 2:** List of the plum rootstock candidates used in this study, their height the leader, internode height of the leader, and trunk diameter of the leader.

Number of Rootstock Candidates	Rootstock Candidates	Internode Height of the Leader (mm)	Height of the Leader (cm)	Trunk Diameter of the Leader (mm)
1	63B78	10.46 r	69 o	5.46 i
2	63B72	9.31 st	82 g	5.23 j
3	Myrobalan 29C	11.21 q	87 cd	6.01 e
4	63B69	7.63 v	43 t	5.06 l
5	63H66	5.35 z	61 r	5.21 j
6	63B11	8.84 u	58 s	6.11 d
7	63B14	7.61 v	66 pq	5.88 f
8	63B16	5.41 z	41 u	4.74 n
9	63B62	6.66 w	37 v	5.78 g
10	63B63	6.22 x	35 w	7.20 a
11	63B61	5.86 y	31 x	3.74 u
12	63B33	9.16 t	57 s	6.35 c
13	63B43	9.46 s	82 g	6.49 b
14	63B76	9.27 t	84 f	5.64 h
15	63H65	11.87 p	93 b	4.77 n
16	63B68	14.07 h	97 a	5.10 k
17	63B60	12.14 o	85 ef	3.91 t
18	63B66	12.79 l	86 de	4.54 o
19	63B13	14.33 fg	82 g	4.75 n
20	63B34	14.24 g	81 gh	5.24 j
21	63B77	15.36 e	88 c	5.12 k
22	63B70	15.80 d	84 f	4.91 m
23	63H30	15.48 e	80 hi	3.75 u
24	63H39	15.40 e	77 kl	3.50 w
25	63B45	17.11 a	79 ij	4.55 o
26	63H46	16.40 c	82 g	4.24 q
27	63B19	14.41 f	81 gh	5.11 k
28	63B12	13.63 i	78 jk	5.24 j
29	63B56	16.83 b	79 ij	4.35 p
30	63B42	13.36 j	76 lm	3.97 t
31	63B53	13.45 j	79 ij	4.12 r
32	63B47	13.18 k	75 mn	3.24 x
33	63B54	13.30 jk	74 n	3.52 vw
34	63H27	13.64 i	69 o	3.78 u
35	63B46	12.06 o	66 pq	4.24 q
36	63B51	12.57 m	67 p	4.51 o
37	63B73	12.83 l	70 o	3.94 t
38	63H35	12.35 n	66 pq	3.72 u
39	63B15	12.33 n	65 q	3.57 v
40	63B18	13.74 i	70 o	4.04 s

Values marked with the same small letter do differ significantly based on Duncan’s test (*p* ≤ 0.05).

**Table 3:** Base sequences of primers used in ISSR analysis, and the total number of bands (TBN), number of polymorphic bands (PBN), and polymorphism ratio (PR) values for each primer pair of rootstock candidates used in the study.

Primers	5′ -> 3′ Base Sequences of Primers	TBN	PBN	PR (%)
DBDACA_7_	DBDACACACACACACACA	12	11	91.7
VHVGTG_7_	VHVGTGTGTGTGTGTGTG	15	15	100.0
GACA_4_	GACAGACAGACAGACA	7	6	85.7
CAC_3_GC	CACCACCACGC	8	2	25.0
BDBCA_7_C	BDBCACACACACACACAC	5	4	80.0
CT_8_TG	CTCTCTCTCTCTCTCTTG	7	7	100.0
GT_6_GG	GTGTGTGTGTGTGG	5	5	100.0
AG8T	AGAGAGAGAGAGAGAGT	7	7	100.0
AGC_6_G	AGCAGCAGCAGCAGCAGCG	5	5	100.0
CA_6_AC	CACACACACACAAC	3	3	100.0
CAB_12_	CABCABCABCABCABCABCABCABCABCABCAB	5	5	100.0
TCC_5_RY	TCCTCCTCCTCCTCCRY	2	2	100.0
GAT_7_C	GAT GATGATGATGATGATGATC	-	-	-
GT8TG	GTGTGTGTGTGTGTGTTG	-	-	-
TAA_8_	TAA TAATAATAATAATAATAATAA	-	-	-
GAA_6_	GAAGAAGAAGAAGAAGAA	-	-	-
Total		81	72	88.9

## Data Availability

Not applicable.

## Supplementary Materials

The following supporting information can be downloaded at: https://www.mdpi.com/article/10.3390/life13071476/s1, Figure S1: This study included selections from the autochthonous germplasm, representing the natural populations of *P. cerasifera* Ehrh., from the Middle Euphrates, Turkey.; Table S1: List of the green plum rootstock candidates used in this study, their height of the leader, internode height of the leader and trunk diameter of the leader for second year.
