# Supplementary material for: Selection and Molecular Characterization of Promising Plum Rootstocks (Prunus cerasifera L.) among Seedling-Origin Trees
Source: Life (Basel). 2023 Jun 29;13(7):1476. doi: 10.3390/life13071476 (PMC10381345; doi:10.3390/life13071476)
Supplement: Supplementary file 1 [file life-13-01476-s001.zip › life-2460340-supplementary.pdf]

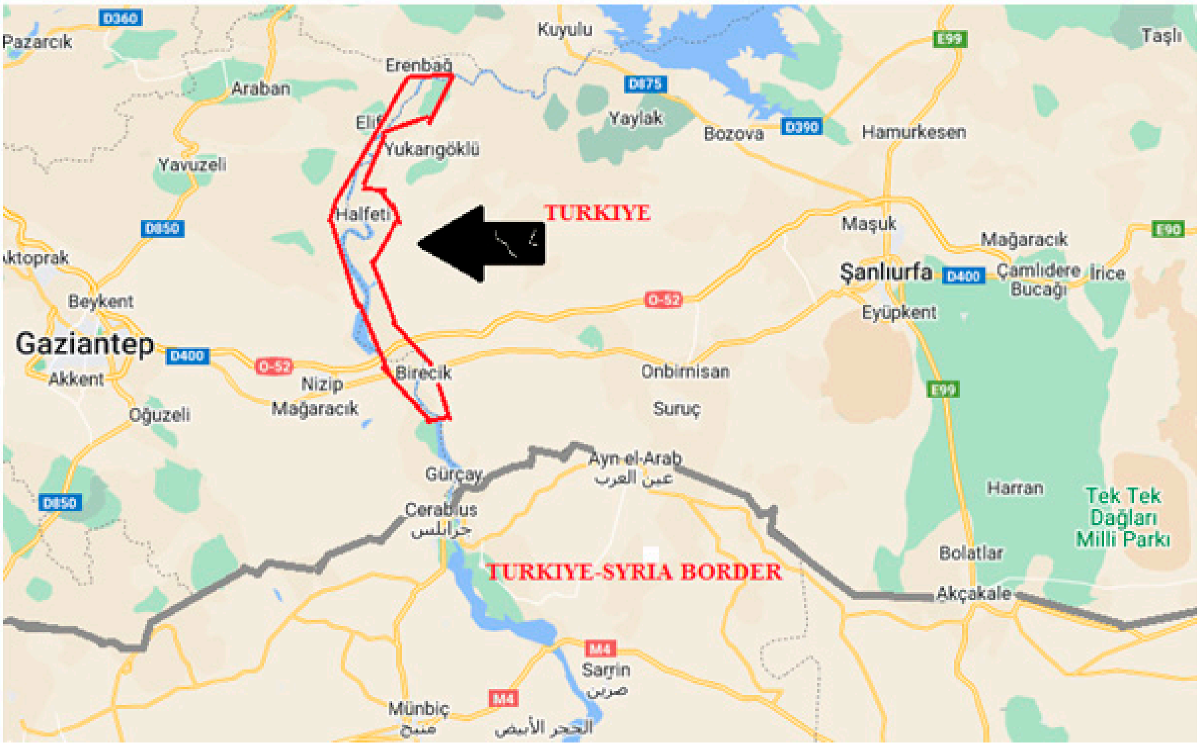

Supplementary Figure S1. This study included selections from the autochthonous germplasm, representing the natural populations of *P. cerasifera* Ehrh., from the Middle Euphrates, Turkey.

Supplementary Table S1. List of the green plum rootstock candidates used in this study, their height of the leader, internode height of the leader and trunk diameter of the leader for second year.

Number of Rootstock candidates	Rootstock candidates	Internode height of the leader (mm)	Height of the leader (cm)	Trunk diameter of the leader (mm)
1	63B78	25.00h	130.33gh	12.30a-f
2	63B72	25.00h	154.66fg	14.01ab
3	Myro29C	25.00h	194.33b-e	15.32a
4	63B69	19.00i	107.00h-j	12.44a-e
5	63H66	15.66jk	171.00ef	12.90a-d
6	63B11	18.00ij	118.33hi	13.88a-c
7	63B14	15.66jk	130.33gh	13.84a-c
8	63B16	14.00k	106.33h-j	11.90b-g
9	63B62	18.00ij	100.33h-j	13.73a-c
10	63B63	16.00i-k	90.66ij	12.81a-d
11	63B61	14.00k	74.33j	8.85g-k
12	63B33	18.00ij	113.00hi	12.44a-e
13	63B43	18.33ij	156.00fg	15.21a
14	63B76	18.33ij	163.33ef	13.25a-d
15	63H65	30.66e-g	233.33a	10.46d-i
16	63B68	33.66b-e	228.00a	10.26d-j
17	63B60	33.33b-e	217.33ab	9.53e-k
18	63B66	30.66e-g	210.66a-c	9.50e-k
19	63B13	29.66g	204.66a-d	9.40e-k
20	63B34	32.00e-g	192.66b-e	9.33e-k
21	63B77	33.66b-e	189.66b-e	9.13f-k
22	63B70	35.33a-d	187.00b-f	8.80g-k
23	63H30	36.33ab	186.66b-f	8.63h-k
24	63H39	36.00ab	186.66b-f	8.36h-k
25	63B45	32.00e-g	186.66b-f	8.33h-k
26	63H46	36.33ab	186.66b-f	8.26h-k
27	63B19	32.33d-g	185.33b-f	8.16h-k
28	63B12	32.00e-g	184.66b-f	8.16h-k
29	63B56	32.00e-g	183.66c-f	8.03h-k
30	63B42	32.00e-g	183.00c-f	7.70h-k
31	63B53	31.00e-g	182.66c-f	7.60h-k
32	63B47	32.33d-g	182.33c-f	7.53i-k
33	63B54	32.00e-g	182.33c-f	7.36i-k
34	63H27	35.66a-c	178.66c-f	7.33i-k
35	63B46	32.00e-g	177.66d-f	7.20jk
36	63B51	32.00e-g	175.00d-f	7.20jk
37	63B73	32.66c-f	173.66d-f	7.13jk
38	63H35	32.00e-g	172.33d-f	7.10jk
39	63B15	32.00e-g	171.00ef	7.10jk
40	63B18	32.00e-g	163.33ef	7.03jk

Values marked with the same small letter do differ significantly based on Duncan's Multiple Range test ($p < 0.05$).

These results were presented to compare the dwarfing characteristics of 39 rootstock candidates and Myro29C clone seedlings for both the first and second year. Our findings showed that seedlings with

dwarfism traits in the first year showed the same character in the second year. For second year, the mean number of characteristics dwarfing associated with in different rootstock candidates, height of the leader, internode height of the leader and trunk diameter of the leader are presented in Supplementary Table S1. 63B16 and 63B61 had the lowest internode height of the leader, followed by 63H66, 63B14 and 63B63. Results indicated that 14 out of 40 rootstock candidate’s internode characters measured showed value smaller than 26 mm compared to other 26 rootstock candidates. In contrast, other 26 rootstock candidates showed values higher than 30 mm. Height of the leader of rootstock candidates ranged from 74.33 to 233.33cm. The studied rootstock candidates showed large differences in height of the leader, so that vigour of rootstock candidates was usually found to be strong (26), intermediate (4), and weak (10). The range of 7.03- 14.01 mm was recorded for trunk diameter of the leader. The rootstock candidates were clustered into four groups based on trunk diameter of the leader including extremely narrow (11 rootstock candidates under 8 mm), narrow (9 rootstock candidates under 9 mm), wide (7 rootstock candidates under 11 mm), wider (13 rootstock candidates over 11 mm). In addition, there were no significant differences between the 11 rootstock candidates and the Myrobolan 29C in terms of the stem diameter of the leader, and these rootstock candidates had similar stem diameter characteristics Supplementary Table S1.
